# Influence of Zinc and Humic Acids on Dye Adsorption from Water by Two Composts

**DOI:** 10.3390/ijerph20075353

**Published:** 2023-03-31

**Authors:** Remigio Paradelo, Paula García, Alba González, Khaled Al-Zawahreh, Maria Teresa Barral

**Affiliations:** 1 CRETUS-Department of Soil Science and Agricultural Chemistry, University of Santiago de Compostela, 15782 Santiago de Compostela, Spain; 2 Department of Earth Sciences and Environment, Prince El-Hassan bin Talal Faculty for Natural Resources and Environment, The Hashemite University, Zarqa 13133, Jordan

**Keywords:** pine bark compost, municipal solid waste compost, basic violet 10, acid blue 113, direct blue 71, adsorption

## Abstract

Searching for alternative low-cost biosorbents for the removal of textile dyes from wastewater is currently an important subject of research. In this work, we have investigated how the presence of other contaminants in textile wastewaters can affect dye adsorption by biosorbents. We tested the adsorption of three dyes of different types: Basic Violet 10 (BV10), Acid Blue 113 (AB113) and Direct Blue 71 (DB71) by two different composts—municipal solid waste compost and pine bark compost—in the presence of Zn (5 mg L^−1^) or dissolved organic matter (100 mg humic acids L^−1^) in batch experiments. Dye adsorption capacity for both composts followed the following sequence: BV10 > AB113 > DB71. In general, dye sorption at the equilibrium was adequately described by the Freundlich model, but not always by the Langmuir model, which did not allow for the estimation of maximum retention capacities in all cases. In general, these were around 1 mg g^−1^ for DB71, 2 mg g^−1^ for AB113, and 40 mg g^−1^ for BV10. Municipal solid waste compost had slightly higher affinity than pine bark compost for the anionic dyes AB113 and DB71, whereas for the cationic dye BV10, pine bark compost presented a much higher adsorption capacity (41.7 mg g^−1^ versus 6.8 mg g^−1^). The presence of Zn or dissolved organic matter in the solutions at typical wastewater concentrations did not decrease the dye adsorption capacity of the composts. This result is positive both for the real application of composts to real textile wastewaters and for the validity of the results of biosorbent performance obtained with single-dye solutions.

## 1. Introduction

The presence of organic dyes in waters represents a major environmental concern for the textile industry because they are highly visible, which greatly affects the perception of water quality by the public, and some types are toxic for organisms [1]. However, many dyes are not readily degraded under the aerobic conditions of biological treatment plants, and other technologies must be used for their elimination, such as electrocoagulation, biodegradation, and others [2]. Among these technologies, low costs of operation and ease of design have made adsorption as a good technology for their removal from water [3,4]. Although activated carbon would be the ideal adsorbent for a wide range of compounds, its high cost has encouraged research for alternative materials as substitutes. Among these, abundant research is being developed around biosorbents, natural materials of different origins, especially of residuals, which can be available in large amounts [4,5,6,7].

In this line of search for low-cost biosorbents, increasing attention has been addressed in recent years to the use of composted wastes [8,9,10,11,12]. Given that they can be produced using a wide range of organic wastes, which otherwise would be landfilled, incinerated, or dumped, composts present interesting characteristics for this use in comparison to other adsorbents, in particular their low production cost and high availability. This low cost in comparison to other adsorbents means that they do not necessarily need to be regenerated and reused several times, so after use they can be discarded or even composted to remove the adsorbed dyes [13]. In this sense, promising results have been presented in the studies that have dealt with textile dye removal from solution by composts so far [8,12,14,15,16,17]. Previous studies from our team have assessed different aspects of the use of compost biosorbents for dye removal, including competitive and noncompetitive removal of different types of dyes, in batch and continuous flow conditions, as well as the effect of other factors such as particle size, solid:liquid ratio, solution pH, or salinity on dye removal, as well as competition with other dyes or continuous flow versus batch conditions [14,15,16,17].

However, some aspects of this application still need to be studied. In most cases, published works have assessed the capacity of composts for dye removal using aqueous solutions of single dyes. However, the presence of other pollutants in wastewaters could potentially interfere with the performance of biosorbents for dye removal. This question is important not only for assessing results in conditions closer to real wastewaters, but also to know if the results obtained from single-dye solutions are comparable or applicable to the treatment of real complex wastewaters. In this context, here we studied dye adsorption by compost in the presence of zinc or dissolved organic matter, which are often presently in textile wastewaters [18], in order to determine the influence of these factors and to assess the validity of the data obtained in laboratory with single aqueous solutions.

## 2. Materials and Methods

### 2.1. Adsorbates

Three textile dyes, each of one class (basic, acid, and direct), were used (Table 1). Basic Violet 10 (BV10 or rhodamine B) was purchased from Panreac (Barcelona, Spain), whereas Acid Blue 113 (AB113) and Direct Blue 71 (DB71) were purchased from Sigma–Aldrich^®^ chemicals (Sigma–Aldrich, St. Louis, MO, USA) with purity 98%.

### 2.2. Biosorbents

In this work we employed two composts produced in composting facilities located in the region of Galicia (northwestern Spain). The first is a municipal solid waste compost (MSWC), obtained at the Complexo Medioambiental do Barbanza (A Coruña, Spain) by large-scale aerobic treatment of the source-separated organic fraction of municipal solid waste. The second one is a composted pine bark (CPB), supplied by the company Costiña Orgánica (A Coruña, Spain), produced by aerobic composting of pine bark in windrows. Properties of the composts are summarized in Table 2. Selection of these specific composts for potential use as biosorbents is due to their limitations to the first-choice application, which would be organic amendments for agronomic use. In this sense, CPB is very acidic and presents very low nutrient levels, while MSWC presents high salinity and Pb and Cu contents. Regarding their surface and spectral properties, studies of N_2_ adsorption–desorption have shown that both materials exhibit type-II isotherms, according to IUPAC classification, which indicate the predominant mesoporous-to-macroporous structure of the adsorbents. Specific surface area of adsorbents, obtained on multipoint BET analysis, is 5.8 and 22.4 m^2^ g^−1^ for MSWC and CPB, respectively (Table 2). Macroporous structure of both composts is confirmed by pore size analysis, with average diameters of 33 Å for MSWC and 90 Å for CPB [15]. The FTIR spectra of the composts (Supplementary Materials) present wide bands in the regions 1000 cm^−1^ and 1600 cm^−1^, corresponding to functional groups potentially involved in dye retention. The peaks at 1050–1030 cm^−1^ are assigned to C-O stretching of carbohydrates, polysaccharides, and alcohol and sulfoxide groups. The bands around 1500–1600 cm^−1^ can be attributed to C=C in the aromatic rings of lignin and lignocellulose and amide and carboxylate group. Further information about the properties of these composts can be found in Paradelo et al. [14] and Al-Zawahreh et al. [15].

### 2.3. Adsorption Experiments

#### 2.3.1. Single-Dye Solutions

For the batch adsorption experiments, we prepared a stock solution of 1000 mg L^−1^ of each single dye in deionized water; this solution was subsequently diluted to the needed concentrations.

The tests for equilibrium adsorption of single dyes were carried out in batch conditions by shaking 0.50 g of each compost with 10 mL of solution with different initial dye concentrations (in the range 0–1000 mg L^−1^ for BV10 adsorption on CPB, and 0–100 mg L^−1^ for the rest of systems) at 130 rpm and 25 °C for 24 h. Afterwards, suspensions were centrifuged for five minutes at 4000× *g* and the clean supernatant was separated for analysis. The dye concentrations (Ce, mg L^−1^) were quantified by measuring absorbance using an UV/VIS spectrophotometer (Varian Cary 100, Agilent Technologies, Inc., Santa Clara, CA, USA) at 547 nm for BV10, 584 nm for DB71 and 566 nm for AB113. Blanks without adsorbent were run in parallel in order to test dye adsorption to the tubes. All tests were carried out in triplicates.

For the study of dye adsorption kinetics, 0.50 g of each compost was put in contact with 10 mL of a solution with 200 mg L^−1^ dye in centrifuge tubes. Suspensions were shaken at room temperature (25 ± 1 °C) on a rotary shaker for different times and afterwards centrifuged at 4000× *g* for 5 min. Aliquots from the supernatant were analyzed immediately, as explained above.

The dye adsorbed by each compost (*q_e_*, mg g^−1^) was calculated as shown in Equation (1):

(1) $$q_{e}=\frac{(C_{o}-C_{e})V}{m}$$

where *V* is the volume of solution (in liters) and *m* is the mass of adsorbent (in grams). Equilibrium adsorption curves were drawn by plotting *C_e_* versus *q_e_*.

For the desorption study, 0.50 g of each compost was suspended in 10 mL of solutions containing 100 mg L^−1^ of each dye individually, shaken, and centrifuged, as explained above. After removing the supernatant, the centrifuged residue was weighed to calculate the amount of dye solution still occluded in the adsorbent. Then, they were resuspended in 10 mL of deionized water, shaken again for 24 h, and centrifuged. Dye concentrations were analyzed in the supernatants as explained above, and the results were expressed as the percentage of desorption of the previously absorbed dye.

#### 2.3.2. Effect of Zn and Dissolved Organic Matter

In order to test the effect of Zn and dissolved organic matter (DOM) on dye removal, equilibrium adsorption curves were built following the general procedure as explained above, but with the addition of a solution of ZnSO_4_ (Panreac, Barcelona, Spain) or humic acids (Sigma-Aldrich, Munich, Germany). The final concentrations in all dye solutions were 5 mg Zn L^−1^ or 100 mg humic acids L^−1^, which were selected as typical concentrations found in real wastewaters [18]. Blanks were run in all cases without dye to determine the absorbance of the Zn or DOM solutions alone. All tests were carried out in triplicates.

### 2.4. Modeling of Adsorption Curves

Equilibrium adsorption was described using the Freundlich and Langmuir isotherms. The Langmuir equation, assuming one monolayer coverage on equal-energy active sites without interaction of the adsorbed solutes, has the following form:

(2) $$q_{e}=\frac{q_{m}K_{L}C_{e}}{1+K_{L}C_{e}}$$

where *q_m_* (mg g^−1^) is the maximum sorption capacity and *K_L_* (L mg^−1^) is the adsorption constant. The expression of the Freundlich isotherm model defines the heterogeneity of the surface as well as the exponential distribution of the active sites and energies:

(3) $$q_{e}=K_{F}C_{e}{}^{1/n}$$

where *K_F_* (L^n^ mg^1−n^ g^−1^) is the Freundlich constant and can indicate uptake capacity, while 1/*n* (dimensionless) measures favorability of the process.

Modeling of the adsorption curves was performed using nonlinear regression analysis. The adsorption models were fitted to the experimental data using the R statistical software version 3.6.1 [19] and the R Commander [20] and nlstools packages for R (The R Foundation for Statistical Computing, Vienna, Austria) [21], which were also used to calculate the significance and quality of the model fits.

## 3. Results and Discussion

**Figure 1:**
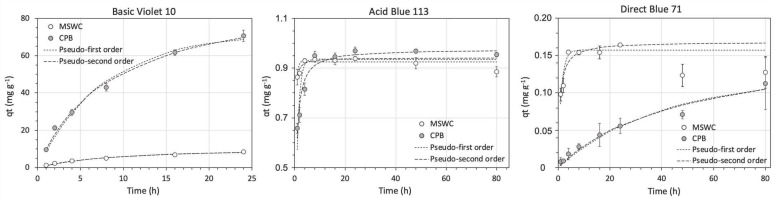
Kinetics of dye adsorption by the composts. Initial dye concentration: 200 mg L^−1^; solid:liquid ratio 1:20MSWC: municipal solid waste compost; CPB: composted pine bark; BV10: Basic Violet 10; AB113: Acid Blue 113; DB71: Direct Blue 71.

The effect of contact time on dye adsorption onto the composts is shown in Figure 1. Equilibrium was more rapidly achieved for AB113 than for the other dyes, in only 4–8 h, and also for the compost MSWC with respect to CPB. The results of modeling are included in the Supplementary Materials.

**Figure 2:**
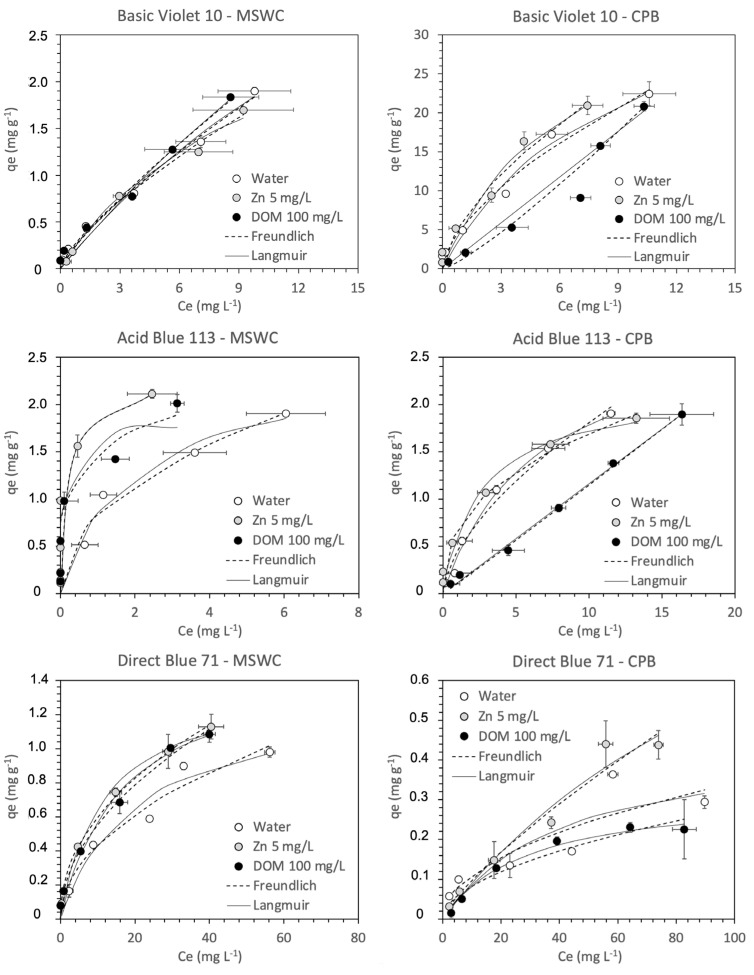
Sorption isotherms of dyes with and without Zn or dissolved organic matter (DOM). MSWC: municipal solid waste compost; CPB: composted pine bark.

The dye adsorption curves with and without Zn or DOM are shown in Figure 2, while the results of the adsorption modeling are presented in Table 3. Both composts had higher sorption capacity for the basic dye than for the rest of types, with an overall sequence of adsorption BV10 >> AB113 > DB71. This significantly higher affinity of compost towards basic dyes is a common observation in the literature [11]. For the dye BV10, the compost CPB was clearly superior to MSWC, whereas MSWC showed a slightly higher adsorption than CPB for the other two dyes. Overall, the Freundlich model was more adequate to describe the experimental data than the Langmuir model, so maximum adsorption capacities could only be calculated confidently in some cases. In general, these were around 1 mg g^−1^ for DB71, 2 mg g^−1^ for AB113, and 40 mg g^−1^ for BV10.

When the composts are compared with other materials used as sorbents, their overall performances are different depending on the type of dye considered. The maximum sorption capacity for the basic dye BV10 is in the same range as those obtained for more expensive synthetic adsorbents such as graphene oxides and activated carbon [22,23], and natural adsorbents such as zeolite and kaolinite [22], citric peels [24] or other composts [25]. In the case of anionic dyes (direct and acid), both composts presented lower affinities than other biosorbents in the literature, as, for example, peels from *Cucumis sativus* [26] or potato [27] for AB113 adsorption. For DB71, the values obtained here are also lower than those reported for other biosorbents such as synthetic alumina/silica oxides [28,29], wheat shells [30], or chitosan/SiO_2_/carbon nanotubes [31], zeolites [32], or active carbon [33]. These results confirm that composts are less competitive as biosorbents for anionic than for cationic dyes, as suggested in a previous work with an exhaustive study of adsorption of different dye classes [15].

**Figure 3:**
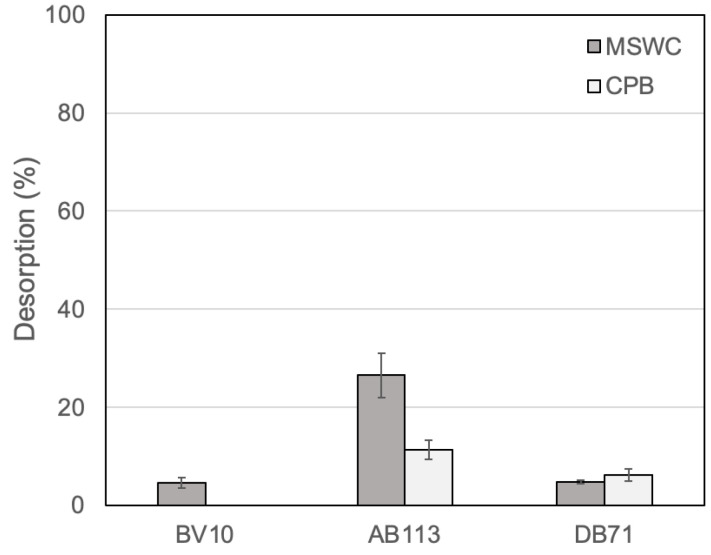
Desorption of the previously adsorbed dye from compost (mean ± standard deviation, n = 3). CPB: composted pine bark; MSWC: municipal solid waste compost; BV10: Basic Violet 10; AB113: Acid Blue 113; DB71: Direct Blue 71.

With the objective of assessing the potential recycling of the composts, desorption of dyes was also studied. The percentages of desorption for the three dyes were very low, as shown in Figure 3, without differences observed between the two composts for DB71. In turn, desorption was lower from CPB than from MSWC in the case of BV10 and AB113 (values under 10% in most cases, with no desorption of BV10), which indicates a stronger interaction of these two dyes with CPB. Overall, the results of the desorption experiment agree with the relative dye adsorption capacities of the composts as has been observed in the studies of equilibrium adsorption.

**Figure 4:**
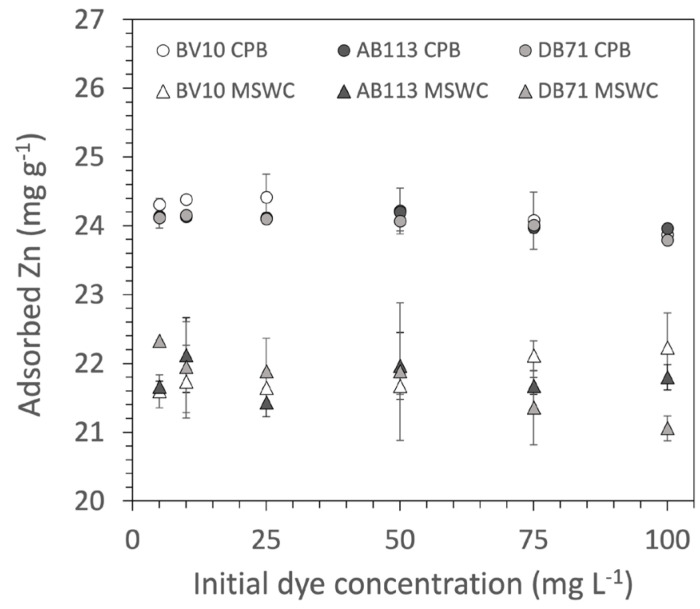
Adsorption of Zn by the composts as a function of initial dye concentrations in the solution (mean ± standard deviation, n = 3). CPB: composted pine bark; MSWC: municipal solid waste compost; BV10: Basic Violet 10; AB113: Acid Blue 113; DB71: Direct Blue 71.

Regarding the influence of other pollutants on adsorption, the comparison of the results in the absence and presence of Zn or DOM have shown the absence of any effect, at least at the concentrations tested here. The presence of Zn in the solution did not modify the dye adsorption curves. Some authors have suggested that Zn could have an antagonistic effect on cationic dye removal [34,35] due to electrostatic interaction competition, since Zn and cationic dyes are positively charged in solution. Given that composts have a high affinity for Zn [36], in theory a high competition with the dyes should have been observed. However, this was not the case, as seen in Figure 4, which shows clearly that the presence of dye at different concentrations did not modify Zn adsorption. A likely explanation would be that the adsorption of Zn and dyes takes place by different mechanisms. Dye adsorption by composted materials is a very complex process, and it is likely that the nature of this interaction cannot by explained by a single mechanism. In this sense, previous works where we investigated the influence of pH and salinity on dye removal, as well as the study of FTIR spectra of dye-loaded composts, have allowed important information to be obtained about the mechanisms of interaction of composts with dyes of different classes [14,15]. These have shown that although electrostatic interaction is an important mechanism of adsorption for both cationic and anionic dyes on compost, it is not the main mechanism, and that a relevant contribution of other mechanisms should be taken into account to explain dye adsorption, for example hydrophobic interactions and dipole–dipole forces [14,15]. If electrostatic interaction is not a major mechanism for adsorption, then Zn is not expected to influence much dye adsorption. Thus, in the experiment presented here, probably hydrophobic interactions are more important for dye adsorption, and Zn does not have an effect in this process. The results obtained in the present work provide additional evidence that electrostatic interaction is not the only mechanism in dye adsorption, as discussed in previous works.

The situation in the case of DOM should be different since the mechanisms for adsorption of the humic acids employed is expected to be similar to the organic dyes. From this point of view, a competition effect would have been expected. Some authors, such as Zermane et al. [37], suggest in turn that the addition of humic acids to the solution should favorably influence the adsorption of basic dyes. However, no effect at all was observed here in most cases, only modifications of the shape of the curve for AB113 with MSWC.

In conclusion, the results of this experiment showed that the presence of other pollutants at typical wastewater concentrations did not significantly modify the dye adsorption capacity of the composts. This does not mean that Zn or DOM cannot influence positively or negatively dye adsorption, but rather that the concentrations at which they are typically found in wastewater, such as those tested here, are too low to have an effect. However, the importance of this fact should not be underestimated: this suggests that the results obtained in laboratory tests using water solutions would be similar to those with real wastewaters, and that the performance of composts for dye removal under real conditions would not be affected by the presence of other pollutants.

## 4. Conclusions

This work evaluated the influence of the presence of common pollutants in textile wastewaters on the performance of compost for dye removal. A pine bark compost and a municipal solid waste compost were tested for the removal of three types of dyes from aqueous solutions—a basic dye (BV10), an acid dye (AB113), and a direct dye (DB71)—with Zn and DOM as the competing pollutants studied. Dye adsorption capacity followed the sequence BV10 > AB113 > DB71, with a slightly higher affinity of municipal solid waste compost for AB113 and DB71, and a much higher adsorption capacity of pine bark compost for BV10. The results of the experiments do not show a significant negative influence of the presence of Zn or DOM with respect to the results in pure dye solutions, suggesting that the presence of these pollutants at typical wastewater concentrations does not modify the adsorption capacity of the composts.

## Figures and Tables

**Table 1:** Main properties of the three dyes.

Dye	Molecular Formula	Structure	Molecular Weight (g mol^−1^)	Water Solubility (g L^−1^)
Basic Violet 10	C_28_H_31_ClN_2_O_3_	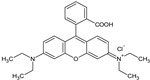	478.5	8–15
Acid Blue 113	C_32_H_21_N_5_Na_2_O_6_S_2_	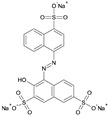	681.7	40
Direct Blue 71	C_40_H_23_N_7_Na_4_O_13_S_4_	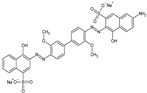	1029.9	10

**Table 2:** Physicochemical parameters of the composts. CPB: composted pine bark; MSWC: municipal solid waste compost; pHw: pH in water; pH_PZC_: pH at point of zero charge; EC: electrical conductivity; OM: organic matter; AEC: anionic exchange capacity; CEC: cation exchange capacity; SSA: specific surface area.

	CPB	MSWC
pH_W_	5.3	8.5
pH_PZC_	4.4	8.1
EC, dS m^−1^	0.4	7.0
OM, %	91.4	41.8
C, %	53.1	24.3
N, %	0.28	1.6
C/N ratio	194	16
CEC, cmol(+) kg^−1^	25.4	18.8
AEC, cmol(−) kg^−1^	4.6	3.6
SSA, m^2^ g^−1^	22.4	5.8

**Table 3:** Isotherm parameters for dye sorption under different conditions; BV10: Basic Violet 10; AB113: Acid Blue 113; DB71: Direct Blue 71; CPB: composted pine bark; MSWC: municipal solid waste compost; DOM: dissolved organic matter; *q_m_*_:_ Langmuir maximum capacity (mg g^−1^); *K_L_*: Langmuir constant (L mg^−1^)_;_ *K_F_*: Freundlich constant (L^n^ mg^1−n^ g^−1^); 1/n: Freundlich coefficient. Significance of the parameter estimations is indicated as follows: * significant at a *p*-value of 0.05; ** significant at a *p*-value of 0.01; *** significant at a *p*-value of 0.001.

Dye	Compost	Factor	Langmuir					Freundlich				
			*K_L_*	*p*	*q_m_*	*p*	R^2^	*K_F_*	*p*	1/n	*p*	R^2^
BV10	CPB	-	0.11 ± 0.05	0.08	41.7 ± 10.2 *	0.01	0.985	5.2 ± 0.9 **	0.004	0.63 ± 0.08 **	0.002	0.979
		Zn	0.16 ± 0.09	0.14	39.3 ± 12.1 *	0.03	0.981	6.2 ± 1.1 **	0.005	0.62 ± 0.11 **	0.004	0.980
		DOM	-	-	-	-	-	1.3 ± 0.2 *	0.01	1.20 ± 0.08 ***	0.0006	0.935
	MSWC	-	0.04 ± 0.03	0.25	6.8 ± 3.9	0.16	0.992	0.31 ± 0.04 **	0.001	0.78 ± 0.06 ***	0.0002	0.993
		Zn	0.10 ± 0.04	0.06	3.4 ± 0.8 *	0.01	0.985	0.33 ± 0.04 **	0.001	0.72 ± 0.06 ***	0.0003	0.988
		DOM	0.03 ± 0.03	0.46	10.4 ± 10.9	0.39	0.991	0.30 ± 0.06 **	0.006	0.83 ± 0.10 **	0.001	0.990
AB113	CPB	-	0.16 ± 0.03 **	0.007	3.0 ± 0.3 ***	0.0004	0.993	0.45 ± 0.06 **	0.002	0.61 ± 0.06 ***	0.0007	0.981
		Zn	0.38 ± 0.15	0.07	2.2 ± 0.3 **	0.001	0.990	0.70 ± 0.10 **	0.002	0.39 ± 0.07 **	0.004	0.988
		DOM	-	-	-	-	-	0.11 ± 0.02 **	0.002	1.01 ± 0.05 ***	0.00005	0.997
	MSWC	-	0.51 ± 0.20	0.07	2.4 ± 0.4 **	0.003	0.977	0.8 ± 0.1 **	0.002	0.47 ± 0.09 **	0.007	0.972
		Zn	4.6 ± 8.5	0.62	2.3 ± 0.9	0.06	0.859	1.8 ± 0.4 *	0.01	0.18 ± 0.27	0.54	0.859
		DOM	11.0 ± 10.3	0.62	1.8 ± 0.3 **	0.004	0.910	1.5 ± 0.2 **	0.002	0.21 ± 0.12	0.14	0.937
DB71	CPB	-	0.02 ± 0.03	0.43	0.5 ± 0.2	0.12	0.765	0.04 ± 0.03	0.26	0.49 ± 0.19	0.06	0.772
		Zn	0.007 ± 0.006	0.29	1.3 ± 0.7	0.16	0.964	0.02 ± 0.01	0.12	0.78 ± 0.13 **	0.004	0.962
		DOM	0.034 ± 0.008 *	0.01	0.32 ± 0.03 ***	0.0004	0.988	0.02 ± 0.01	0.07	0.53 ± 0.10 **	0.007	0.945
	MSWC	-	0.05 ± 0.02	0.09	1.3 ± 0.2 **	0.005	0.964	0.14 ± 0.04 *	0.02	0.50 ± 0.07 **	0.003	0.965
		Zn	0.09 ± 0.02 *	0.02	1.4 ± 0.1 ***	0.0005	0.993	0.21 ± 0.03 **	0.001	0.46 ± 0.04 ***	0.0002	0.994
		DOM	0.06 ± 0.02 *	0.04	1.5 ± 0.2 **	0.002	0.991	0.17 ± 0.03 **	0.003	0.50 ± 0.05 ***	0.0004	0.993

## Data Availability

The data presented in this study are available on request from the corresponding author.

## Supplementary Materials

The following supporting information can be downloaded at: https://www.mdpi.com/article/10.3390/ijerph20075353/s1, Figure S1: FTIR spectra of the composts. MSWC: municipal solid waste compost; CPB: composted pine bark. Table S1: Kinetic parameters for pseudo-first and pseudo-second order models for dyes sorption rates by composts. CPB: composted pine bark; MSWC: municipal solid waste compost; *q_e_*(*model*)**: equilibrium sorption value predicted from the model (mg g^−1^); *k_1_*: pseudo-first order model constant (h^−1^); *k_2_*: pseudo-second order model constant (g mg^−1^ h^−1^).
