# Supplementary material for: Influence of Zinc and Humic Acids on Dye Adsorption from Water by Two Composts
Source: Int J Environ Res Public Health. 2023 Mar 31;20(7):5353. doi: 10.3390/ijerph20075353 (PMC10094621; doi:10.3390/ijerph20075353)
Supplement: Supplementary file 1 [file ijerph-20-05353-s001.zip › ijerph-2282099-supplementary.pdf]

Influence of Zinc and Humic Acids on Dye Adsorption from Water by Two Composts

Remigio Paradelo, Paula García, Alba González, Khaled Al-Zawahreh and Maria Teresa Barral

Supplementary material

A. FTIR spectra of adsorbents

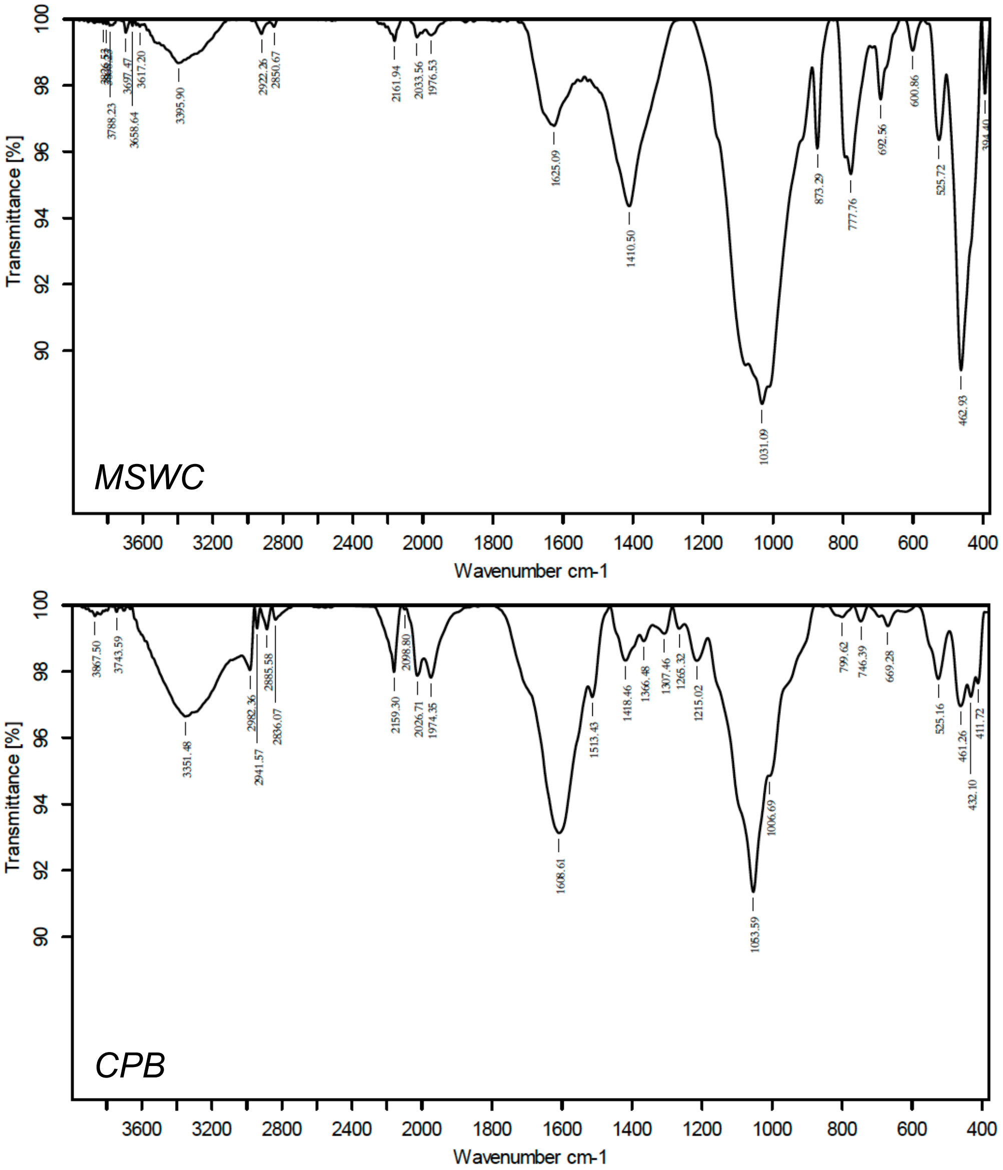

Figure S1. FTIR spectra of the composts. MSWC: municipal solid waste compost; CPB: composted pine bark.

B. Kinetic modelling

Adsorption kinetics data were described using the Lagergren's pseudo-first order and pseudo-second order models, defined by Equations (1) and (2), respectively:

$$q_a = q_e(1 - e^{-k_1 t}) \qquad (1)$$

$$q_a = \frac{k_2\, q_e^2\, t}{1 + k_2\, q_e\, t} \qquad (2)$$

where q_a (mg g^{-1}) is the amount of dye removed by the compost at a time t, q_e (mg g^{-1}) is the amount of dye removed at equilibrium (this parameter is fitted by the model), k_1 (h^{-1}) is the pseudo-first-order rate constant, and k_2 (g mg^{-1} h^{-1}) is the pseudo-second-order rate constant.

Table S1. Kinetic parameters for pseudo-first and pseudo-second order models for dyes sorption rates by composts. CPB: composted pine bark; MSWC: municipal solid waste compost; $q_e(model)$: equilibrium sorption value predicted from the model (mg g^{-1}); k_1: pseudo-first order model constant (h^{-1}); k_2: pseudo-second order model constant (g mg^{-1} h^{-1}).

Dye	Compost	Pseudo-first order model		Pseudo-second order model	
		k_1	$q_e(model)$	k_2	$q_e(model)$
Basic Violet 10	CPB	6.84	78.5	0.90	71.5
	MSWC	5.04	10.1	0.60	10.4
Acid Blue 113	CPB	0.95	0.93	10.9	0.94
	MSWC	2.63	0.92	1.77	0.98
Direct Blue 71	CPB	0.03	0.12	0.12	0.17
	MSWC	0.79	0.16	7.66	0.17
